# Peri-delivery Calcium Administration and Postpartum Bleeding: A Systematic Review and Meta-analysis

**DOI:** 10.1097/og9.0000000000000186

**Published:** 2026-07-30

**Authors:** Julia Romina M. Boers, Mirely Garcia, Fabrizio Zullo, Christian J. Miller, Moeun Son

**Affiliations:** Department of Obstetrics and Gynecology, Division of Maternal–Fetal Medicine, Weill Cornell Medical College, New York, New York; Department of Obstetrics, Amsterdam UMC location Vrije Universiteit, Amsterdam, the Netherlands; Amsterdam Reproduction and Development Research Institute, Amsterdam, the Netherlands; Department of Maternal and Child Health and Urological Sciences, Sapienza University of Rome, Italy; and ILR School/Cornell University Library, Ithaca, New York.

## Abstract

**OBJECTIVE:**

To examine whether peri-delivery administration of exogenous calcium improves postpartum bleeding outcomes.

**DATA SOURCES:**

We conducted a systematic review of randomized controlled trials (RCTs) and quasi-randomized trials comparing pregnant individuals undergoing vaginal or cesarean delivery who received exogenous calcium before or within 1 hour of delivery with those who received no calcium supplementation. We searched Ovid MEDLINE, Ovid EMBASE, CINAHL, Scopus, Web of Science, and CENTRAL/The Cochrane Library (including ClinicalTrials.gov) from inception through July 10, 2025 (PROSPERO 2025 CRD420251091214).

**METHODS OF STUDY SELECTION:**

Titles, abstracts, and full texts were independently screened in duplicate. Six RCTs and one quasi-randomized study including 806 participants (403 intervention, 403 control) were included. All participants underwent cesarean delivery. Interventions included intravenous calcium chloride or calcium gluconate, administered after delivery, after cord clamping, or perioperatively.

**TABULATION, INTEGRATION, AND RESULTS:**

The primary outcome was estimated or quantitative postpartum blood loss. Secondary outcomes included the use of additional uterotonics beyond prophylactic oxytocin and other postpartum bleeding-related outcomes. Random‐effects models were used to calculate ratio of means, pooled mean differences, and risk ratios (RRs) with 95% CIs. Calcium supplementation was associated with 8% lower postpartum blood loss compared with control (ratio of means 0.92, 95% CI, 0.88–0.97). Calcium supplementation was associated with a lower frequency of additional uterotonic use (27.1%) compared with controls (41.9%) (RR 0.69, 95% CI, 0.49–0.96). Low-to-moderate heterogeneity was observed, and most studies were judged to be at low risk of bias.

**CONCLUSION:**

Peri-delivery calcium administration was associated with reduced postpartum blood loss and decreased need for additional uterotonics. Although effect sizes were modest and evidence was limited by few studies and heterogeneity in calcium formulations and doses, these findings suggest that calcium may enhance uterine contractility. Larger RCTs are needed to confirm efficacy and to determine optimal dosing.

**SYSTEMATIC REVIEW REGISTRATION:**

PROSPERO, CRD420251091214.

## Abstract 2

Peri-delivery calcium administration was associated with reduced postpartum blood loss and decreased use of additional uterotonics, suggesting a potential role as a uterotonic adjunct.

Postpartum hemorrhage (PPH) is the leading cause of maternal morbidity and mortality in the United States and globally.^1–3^ Approximately 14 million women are affected each year, resulting in up to 70,000 maternal deaths worldwide.^4^ The most common case is uterine atony, responsible for 70–80% of PPH cases.^5^ Uterine atony is the inability of the uterus to contract adequately after delivery, resulting in continued bleeding. When synthetic oxytocin fails to treat PPH, second-line uterotonics such as methergine, carboprost, and misoprostol are used.^6^ However, they can be more expensive, may cause significant side effects, are contraindicated in some patients, and are not always effective. With rising global rates of PPH attributable to uterine atony, there is a growing need for alternative or adjunct uterotonics.^5,6^

Calcium is essential for initiating and maintaining labor contractions.^7–10^ These contractions are triggered by intracellular calcium influx, which binds to calmodulin and activates a cascade leading to actin–myosin interaction and ultimately uterine muscle contraction.^7,11–13^ In vitro studies show that low extracellular calcium levels and blockade of calcium entry into smooth muscle cells reduce myometrial sensitivity to oxytocin and decrease contractile activity.^14–18^ Observational studies further suggest that higher maternal serum calcium levels may trigger physiologic uterine contractility during labor.^19^ In addition, low levels of ionized calcium have been associated with adverse outcomes, including emergency cesarean delivery, neonatal intensive care admission, increased postpartum blood loss, and greater PPH severity.^20,21^ Beyond its role in uterine contractility, calcium is an important cofactor in the coagulation pathway and is essential for postpartum hemostasis.^20^ Collectively, these data suggest that intrapartum calcium supplementation may enhance uterine contractility, thereby improving delivery outcomes and reducing postpartum blood loss, as well as the risk and severity of PPH. Although some studies suggest reduced blood loss, results remain inconsistent.^22–28^

To date, no systematic review has synthesized the evidence on inpatient calcium administration during the peripartum period as a prophylactic or therapeutic uterotonic agent. Given its potential as an inexpensive, heat-stable, and widely available uterotonic, calcium could serve as a simple, accessible adjunct uterotonic worldwide.^23^ We therefore conducted a systematic review and meta-analysis to evaluate the effect of inpatient exogenous calcium administration during the peripartum period on postpartum bleeding outcomes.

## SOURCES

This systematic review and meta-analysis was conducted in adherence to the PRISMA (Preferred Reporting Items for Systematic Reviews and Meta-analyses) guidelines. The review protocol was registered and published on PROSPERO (CRD420251091214) on July 10, 2025.

A comprehensive search was conducted by a medical librarian (C.J.M.) using PubMed and EMBASE, and it was then translated to other databases, including CINAHL, Scopus, Web of Science, and CENTRAL/The Cochrane Library (including ClinicalTrials.gov). The databases were searched on July 24, 2025, for articles published from inception to July 2025 to identify articles on the use of calcium supplementation peri-delivery and postpartum bleeding outcomes. Relevant subject headings and key words relating to calcium supplementation, delivery, and postpartum blood loss outcomes were used. There were no language, geographic location, or publication date restrictions. The results were deduplicated. The complete search strategy is presented in Appendix 1 (available online at https://links.lww.com/AOG/E765).

## STUDY SELECTION

The screening process was performed in Covidence, a screening and data extraction tool. Two authors (J.R.M.B., M.G.) independently performed title and abstract screening of retrieved studies. The selected full texts were screened against predefined inclusion and exclusion criteria. Studies were eligible for inclusion if they assessed pregnant individuals with live gestations undergoing vaginal or cesarean delivery who received inpatient calcium administration before or within 1 hour of delivery. Any form and dose of calcium, including calcium gluconate and calcium chloride, were accepted. Only data from randomized controlled trials (RCTs) and quasi-randomized trials were included. The control group involved placebo or no calcium supplementation. We planned to exclude nonrandomized or observational studies, animal or in vitro studies, abstracts or proceedings, and nonoriginal data such as reviews, commentaries, or editorials. The reference lists of included studies were cross-checked for additional eligible studies. Any disagreement with regard to the relevance of studies was resolved through discussion or, if needed, consultation of a third party (M.S.).

Data extraction was independently performed by two authors (J.R.M.B., M.G.) to record study characteristics, participant characteristics, and outcomes. Extracted study characteristics included author, publication year, study design, country, sample size, calcium formulation and dose, and eligibility criteria. Participant characteristics included maternal age, gestational age at delivery, body mass index (BMI), prior cesarean delivery, and baseline serum ionized calcium. The primary outcome was postpartum blood loss, assessed by either estimated or quantitative measures. Secondary outcomes included the need for additional uterotonics beyond prophylactic oxytocin (defined as the administration of one or more uterotonic agents [misoprostol, ergometrine, carboprost, methylergometrine, carbetocin] or antifibrinolytic [tranexamic acid]), number of blood transfusions, uterine atony (as defined by authors), PPH (more than 1,000 mL), uterine tone (numerical score or binary outcome), changes in hematologic parameters, postinfusion ionized calcium levels, maternal adverse events such as cardiac arrhythmias and burning or discomfort in the intravenous site, and neonatal outcomes. As specified in the protocol, a broader set of outcomes based on the core outcome set for induction of labor was initially planned; however, because these outcomes were not consistently reported in the included studies, only the secondary outcomes that were available across the studies were extracted and analyzed. Corresponding and first authors of included studies were contacted to inform them of the inclusion of their study in this meta-analysis and to request additional data if available. If no response was received, one reminder was sent.

Two reviewers (J.R.M.B., M.G.) independently rated the methodologic quality of the included studies using RoB 2 (revised Cochrane Risk of Bias tool for RCTs). A third author (M.S.) served as the arbitrator. The RoB 2 evaluates risk of bias across five domains, including bias arising from 1) the randomization process, 2) deviations from intended interventions, 3) missing outcome data, 4) measurement of the outcome, and 5) selection of reported results. According to these judgments, the studies were classified as having low risk of bias, some concerns, or high risk of bias. The certainty of the findings was assessed with the GRADE (Grading of Recommendations Assessment, Development, and Evaluation) approach. Each outcome was evaluated across five domains: risk of bias, inconsistency, indirectness, imprecision, and publication bias. The overall certainty of the evidence was then specified as high, moderate, low, or very low.

Data from the included studies were analyzed with Stata/SE 18.0. When data were reported as medians with interquartile ranges, they were converted to mean±SD provided that the distributions were not clearly skewed.^29^ When data appeared skewed or transformation was not appropriate, values were reported as not transformed.

Baseline characteristics of the study groups were presented descriptively. A total of eight categorical outcomes and seven continuous outcomes were examined; three outcomes investigated the newborn well-being, and 12 outcomes were (adverse) maternal outcomes.

Dichotomous outcomes were pooled as risk ratios (RRs) with 95% CIs. Continuous outcomes with approximately symmetric distributions were pooled as mean differences. Because postpartum blood loss is typically right skewed, this outcome was analyzed with the ratio of means, estimated on the logarithmic scale and back-transformed for interpretation. Random-effects models using restricted maximum likelihood were applied, and heterogeneity was assessed with *I*^2^ and τ^2^.

For studies with multiple intervention arms, data were handled in accordance with Cochrane recommendations to avoid unit‐of‐analysis errors. Specifically, the study conducted by Farber et al^26^ included two intervention arms—calcium chloride 200 mg and calcium chloride 400 mg—compared with a shared placebo group. To prevent double counting of the control group, only the calcium chloride 400-mg arm was included in the meta‐analysis.

Statistical heterogeneity was quantified with both the *I*^2^ statistic and τ^2^, with values interpreted according to standard thresholds.^30^ Statistical significance was defined as *P*<.05.

Predefined subgroup analyses were planned to explore potential differences in outcomes based on parity (nulliparous vs multiparous individuals), mode of delivery (vaginal vs cesarean delivery), labor status (labored vs unlabored deliveries), and oxytocin exposure (with vs without synthetic oxytocin administration for labor induction or augmentation). Moreover, a sensitivity analysis was planned to assess the robustness of the pooled estimates, particularly after exclusion of the two studies judged to be at high risk of bias. However, these analyses were not conducted because of insufficient data and inconsistent reporting of these variables across included studies.

Potential publication biases were planned to be assessed graphically with a funnel plot of the primary outcome and statistically with Begg and Egger tests provided that the number of included studies exceeded 10.

## RESULTS

**Fig. 1.**
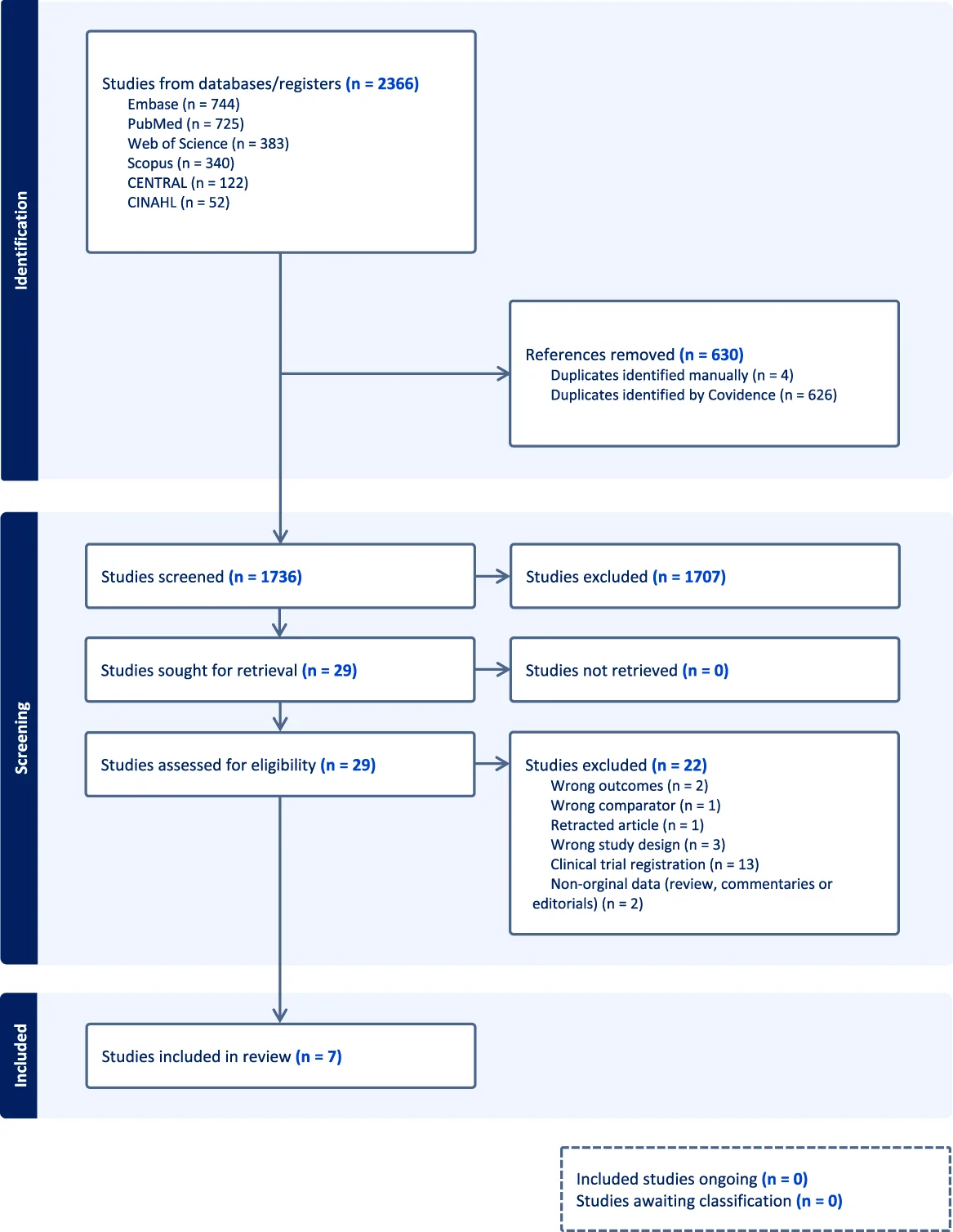
PRISMA (Preferred Reporting Items for Systematic Reviews and Meta-analyses) flow diagram of study selection.

The PRISMA flow diagram is presented in Figure 1. After the removal of duplicates, the initial search yielded 1,736 studies. Of those, 1,707 were excluded after title and abstract screening. A full-text assessment of eligibility was performed on the remaining 29 studies. Subsequently, 22 were excluded for the following reasons: clinical registrations without published full text (n=13), wrong study designs (n=3), wrong outcomes (n=2), nonoriginal data (n=2), retracted articles (n=1), and wrong comparators (n=1). As a result, seven studies were included for analysis.^22–28^

*Boers. Peri-delivery Calcium Administration. O&G Open 2026*.

Six RCTs and one quasi-randomized trial with a total of 806 individuals (403 intervention, 403 control) were included. The characteristics of the included studies are summarized in Table 1. All participants had undergone cesarean delivery. Labor status varied across studies: Five studies included women who had labored before cesarean delivery,^22–25,28^ whereas two trials included only women who underwent unlabored cesareans deliveries.^26,27^ The type and timing of calcium supplementation varied. Interventions included intravenous calcium chloride (four RCTs)^22,23,26,28^ and intravenous calcium gluconate (three RCTs)^24,25,27^ given after delivery or cord clamping (five RCTs)^22–24,26,28^ or perioperatively (two RCTs).^25,27^ All studies included a control group, with six studies using saline placebo^22–27^ and one study using no calcium supplementation.^28^ All participants received standard prophylactic synthetic oxytocin infusion (Pitocin) after delivery, although specific dosing protocols varied by hospital. The studies were conducted in the United States (n=3),^22,23,26^ India (n=3),^24,25,27^ and Pakistan (n=1).^28^ Six of the seven included studies reported the prespecified primary outcome of postpartum blood loss.^22–27^ Only Ansari et al^23^ and Yadav et al^25^ used quantitative blood loss by measuring suction canister contents minus weights of other fluids and surgical pads. The remaining studies used estimated blood loss, assessed by visually examining suction jars and weighing the soaked gauze.^22,24,26^ One study did not provide a description of how postpartum blood loss was measured.^27^

**Table 1.** Description of Included Studies

	Ansari et al,^22^ 2022	Ansari et al,^23^ 2024	Abirami et al,^24^ 2025	Yadav et al,^25^ 2025	Farber et al,^26^ 2015	Agarwal et al,^27^ 2022	Yaqub et al,^28^ 2025
Study location	United States	United States	India	India	United States	India	Pakistan
Design	Double-blind pilot RCT	Double-blind RCT	Double-blind RCT	Double-blind RCT	Double-blind RCT	Double-blind RCT	QRT*
Months of study	14	12	14	NR	19	NR	6
No. of patients†	40 (20 vs 20)	120 (60 vs 60)	367 (183 vs 184)	100 (50 vs 50)	39 (19 vs 20)	60 (30 vs 30)	80 (41 vs 39)
Participants/inclusion criteria	Pregnant patients undergoing CD with at least 2 uterine atony risk factors: intrapartum delivery, oxytocin infusion for 4 h or more, magnesium infusion, chorioamnionitis, multiple gestation, polyhydramnios, or prior PPH	Laboring patients at 34 wk or more who received oxytocin for labor induction or augmentation and subsequently required CD	Pregnant patients undergoing an intrapartum CD (labor with or without oxytocin administration before CD) under spinal anesthesia	Preeclamptic women receiving magnesium sulfate therapy undergoing CD	Healthy women with singleton gestation at 37 wk or more undergoing elective CD	Full-term pregnant patients with singleton pregnancy, without systemic illness, scheduled for CD under spinal anesthesia	Pregnant patients at 38 wk or more at risk of uterine atony or PPH per AWHONN criteria and were subjected to elective or emergency CD
Labor status	Labored	Labored	Labored	Labored and unlabored	Unlabored	Unlabored	Labored and unlabored
Dose and route	1 g IV calcium chloride (=272 mg elemental calcium) over 10 min after cord clamping	1 g IV calcium chloride (=272 mg elemental calcium) over 10 min after umbilical cord clamping	1 g IV calcium gluconate (=93 mg elemental calcium) over 10 min after umbilical cord clamping	500 mg IV calcium gluconate (=46.5 mg elemental calcium) over 15 min before spinal anesthesia	400 mg IV calcium chloride (=109 mg elemental calcium) over 4 min after umbilical cord clamping in combination with 5 units oxytocin	1 g IV calcium gluconate (=93 mg elemental calcium) over 20 min after spinal anesthesia	1 g IV calcium chloride (=272 mg elemental calcium) after umbilical cord clamping
Control	Placebo	Placebo	Placebo	Placebo	Placebo with 5 units oxytocin	Placebo	No treatment
Oxytocin at delivery	All patients received an oxytocin bolus of 2 units followed by an infusion of 7.5 units/h for 4 h	All patients received an oxytocin bolus of 2 units followed by an infusion of 7.5 units/h	All patients received a slow oxytocin bolus of up to 2 units followed by an infusion of 10 units/h	All patients received 10 units of oxytocin added to 200 mL Ringer lactate solution	All patients received IV oxytocin 30 units/L at 100 mL/h for 5 h	All patients received 5 units of oxytocin given slowly IV followed by 5 units along with IV fluid infusion	All patients received oxytocin 10 units IV
Primary outcome‡	Incidence of uterine atony	Quantitative blood loss	Uterine tone	Incidence of postspinal hypotension	Change in blood pressure	Incidence of hypotension	Incidence of uterine atony
Secondary outcomes	Postpartum blood loss, uterine tone numerical rating scores, serial venous blood calcium levels, hemodynamic parameters, and adverse effects	Incidence of PPH, need for additional uterotonic administration, blood transfusion, change in hematocrit, and overall safety and tolerability	Postpartum blood loss, need for additional uterotonic administration, and blood product transfusion	Postpartum blood loss, neonatal outcomes, and maternal morbidity	Heart rate, uterine tone, need for additional uterotonic administration, vasopressor use, and postpartum blood loss	hemodynamic parameters, postpartum blood loss, adverse effects, and neonatal outcomes	NR

RCT, randomized controlled trial; QRT, quasi-randomized trial; NR, not reported; CD, cesarean delivery; PPH, postpartum hemorrhage; AWHONN, Association of Women's Health, Obstetric and Neonatal Nurses; IV, intravenous.

Summary characteristics of trials included in the meta-analysis.

* QRTs were defined as those in which participants were allocated to study arms according to a pseudorandom sequence.

† Farber et al^26^ included three groups: 1) 20 participants who received 200 mg IV calcium chloride, 2) 20 participants who received 400 mg IV calcium chloride, and 3) 20 participants who received a placebo. For this meta-analysis, data from the 400-mg calcium chloride group were used as the intervention group. Data from the 200-mg calcium chloride group were excluded.

‡ Ansari et al^22^ defined the incidence of uterine atony as a binary composite outcome including any of the following: second-line uterotonic requirement; blood loss exceeding 1,000 mL; placement of a Bakri balloon, B-Lynch suture, or O'Leary sutures by the obstetrician; uterine artery embolization; or hysterectomy.

Two authors responded to requests for additional data.^22,23,25^ Yadav provided a deidentified dataset; however, it did not contain additional data suitable for inclusion in the meta-analysis. Ansari provided additional data from the 2022 pilot trial, including postpartum blood loss, use of additional uterotonics, and hematocrit change, and data from the 2024 RCT, including incidence of uterine atony, postinfusion ionized calcium levels, change in ionized calcium levels, and hematocrit change.

Baseline characteristics of the overall study population are shown in Table 2. Maternal characteristics appeared to vary across studies from different countries, with notably lower mean maternal age, BMI, and baseline ionized calcium and more frequent prior cesarean delivery in studies from India and Pakistan compared with those from the United States. However, randomization within each study resulted in comparable groups.

**Table 2.** Baseline Characteristics: Intervention Versus Control Group

	N (403 vs 403)	Age (y)	Gestational Age at Randomization (wk)	Body Mass Index	Prior Cesarean Delivery	Serum Ionized Calcium Before Prophylactic Calcium Administration (mmol/L)
Ansari et al,^22^ 2022	20 vs 20	34.5±6.6 vs 33.5±3.7	38.4 (IQR 36.8–39.3) vs 39.0 (IQR 35.7–39.7)	31±6 vs 30±6	4 (20) vs 3 (15)	1.18 (95% CI, 1.16–1.19) vs 1.18 (95% CI,1.16–1.19)
Ansari et al,^23^ 2024	60 vs 60	33.8±5.2 vs 32.8±5.2	39 (QR 39–40) vs 39 (IQR 39–40)	31.6±4.6 vs 32.2±6.1	5 (8.3) vs 3 (5.0)	NR
Abirami et al,^24^ 2025	183 vs 184	27±4.7 vs 27±4.1	39 (IQR 38–39) vs 39 (IQR 38–39)	26±4.0 vs 26±3.7	35 (19.1) vs 36 (19.5)	0.88±0.14 vs 0.90±0.15
Yadav et al,^25^ 2025	50 vs 50	28.3±5.8 vs 27.1±4.1	37 (range 0–6 d) vs 37 (rage 0−5 d)	NR	NR	NR
Farber et al,^26^ 2015	19 vs 20	35±4.0 vs 34±3.0	38.9±0.6 vs 38.8±0.9	33±7.1 vs 30±2.1	15 (79) vs 20 (100)	1.21±0.06 vs 1.20±0.04
Agarwal et al,^27^ 2022	30 vs 30	25.7±3.5 vs 26.8±7.9	NR	NR	NR	NR
Yaqub et al,^28^ 2025	41 vs 39	29.0±3.4 vs 28.0±3.7	NR	26.0±2.4 vs 26.5±2.4	33 (80.5) vs 33 (84.6)	NR

IQR, interquartile range; NR, not reported.

Data are mean±SD, median (interquartile range or range), or n (%).

**Fig. 2.**
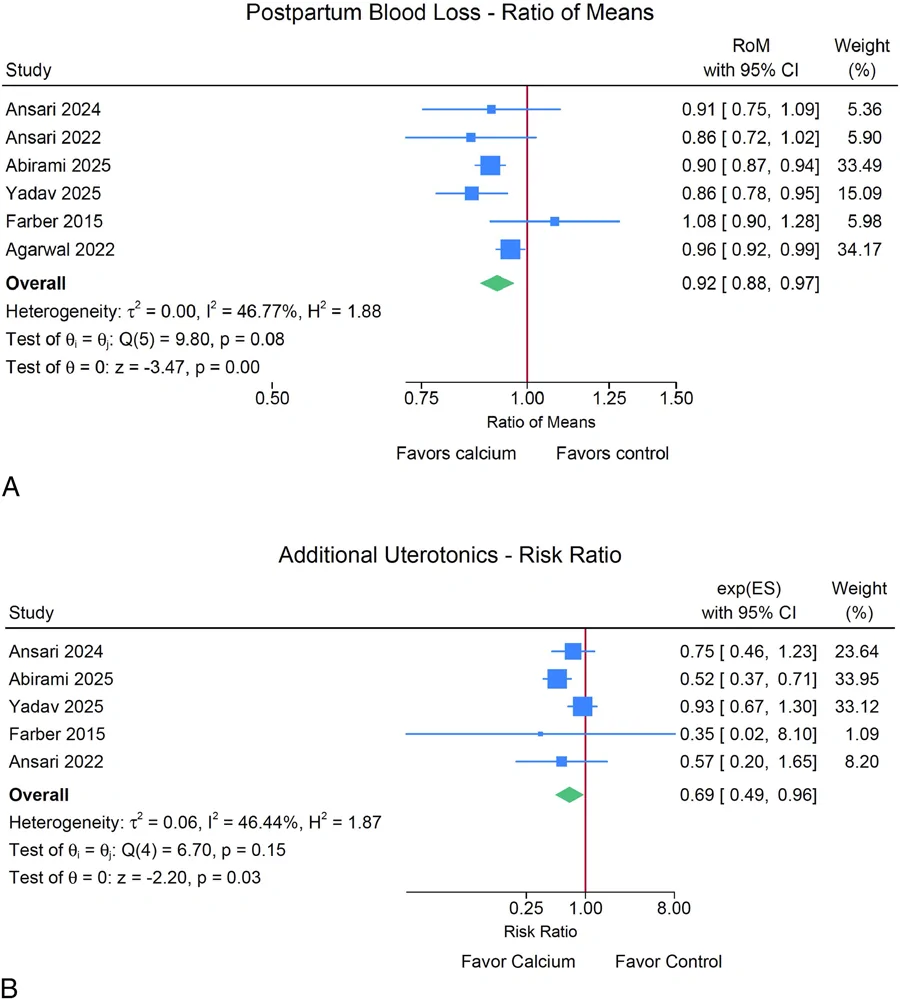
Random-effects meta-analysis of postpartum blood loss **(A)** and use of additional uterotonics beyond prophylactic oxytocin **(B)**. Effect estimates are shown with 95% CIs. RoM, ratio of means.

An overview of maternal outcomes is presented in Table 3. Six studies reported postpartum blood loss and were included in the updated analysis using the ratio of means. Calcium supplementation was associated with 8% lower postpartum blood loss compared with control (ratio of means 0.92, 95% CI, 0.88–0.97, *P*<.001, *I*^2^=46.77%, τ^2^=0.00) (Fig. 2A). Five studies reported the need for additional uterotonics beyond prophylactic oxytocin, involving 666 patients (332 vs 334). All studies demonstrated a reduced need for additional uterotonics compared with control, although not all achieved statistical significance. Overall, calcium supplementation was associated with a lower need for second line uterotonics (27.1% vs 41.9%, RR 0.69, 95% CI, 0.49–0.96, *P*=.027); moderate heterogeneity was observed (*I*^2^=46%, τ^2^=0.0578) (Fig. 2B). Three studies reported the number of patients who received a blood transfusion, involving 527 patients (263 in the intervention group vs 264 in the control group). Calcium supplementation was not associated with decreased risk of receiving a blood transfusion (RR 0.55, 95% CI, 0.23–1.33, *P*=.18, *I*^2^=0%, τ^2^=0.0) (Appendix 2, https://links.lww.com/AOG/E765). The occurrence of maternal cardiac arrhythmias was reported in two studies involving 160 patients (80 vs 80). Calcium supplementation was not associated with increased risk of maternal cardiac arrhythmias (RR 0.48, 95% CI, 0.08–2.69, *P*=.402, I^2^=47%, τ^2^=0.775) (Appendix 2, https://links.lww.com/AOG/E765). Burning or discomfort at the intravenous site was reported in three studies involving 527 patients (263 vs 264). Calcium supplementation was not associated with increased risk of reported burning or discomfort at the intravenous site (RR 0.74, 95% CI, 0.14–3.88, *P*=.73, I^2^=0%, τ^2^=0.00) (Appendix 2, https://links.lww.com/AOG/E765).

**Table 3.** Maternal Outcomes: Intervention Versus Control Group

	Ansari et al,^22^ 2022 (20 vs 20)	Ansari et al,^23^ 2024 (60 vs 60)	Abirami et al,^24^ 2025 (183 vs 184)	Yadav et al,^25^ 2025 (50 vs 50)	Farber et al,^26^ 2015 (19 vs 20)	Agarwal et al,^27^ 2022 (30 vs 30)	Yaqub et al,^28^ 2025 (41 vs 39)	Total	RR or MD or RoM (95% CI; *P*; *I*^2^; τ^2^)
Postpartum blood loss (mL)	735.8±236.8 vs 854.8±389.3	989.3±595.1 vs 1,082.4±454.9	526.0±55.2 vs 581.5±148.9	406.90±94.34 vs 472.20±122.49	634±180 vs 588±161	825.67±59.63 vs 864.00±68.61	NR	NA	**0.92 (0.88 to 0.97); <.001; 46.77%; 0.00**
Additional uterotonics beyond prophylactic oxytocin	4/20 (20.0) vs 7/20 (35.0)	18/60 (30.0) vs 24/60 (40.0)	40/183 (21.7) vs 78/184 (42.4)	28/50 (56.0) vs 30/50 (60.0)	0/19 (0.0) vs 1/20 (5.0)	NR	NR	90/332 vs 140/334	**0.69 (0.49 to 0.96); .027; 46%; 0.0578**
Blood transfusion	1/20 (5.0) vs 1/20 (5.0)	5/60 (8.3) vs 9/60 (15.0)	1/183 (0.6) vs 3/184 (1.6)	NR	NR	NR	NR	7/263 vs 13/264	0.55 (0.23 to 1.33); .18; 0%; 0.0
Incidence of uterine atony	4/20 (20.0) vs 10/20 (50.0)	30/60 (30.0) vs 42/60 (70.0)	NR	NR	NR	NR	7/41 (17.1) vs 15/39 (38.5)	41/121 vs 67/119	**0.60 (0.40 to 0.89); .01; 24%; 0.04**
Incidence of postpartum hemorrhage	NR	24/60 (40.0) vs 34/60 (56.7)	NR	NR	NR	NR	NR	24/60 vs 34/60	NC
Uterine tone* after 10 min after infusion	NT	7.0±1.51 vs 7.0±1.51	NT	NR	8.5±0.9 vs 8.7±1.0	NR	NR	NA	−0.09 (−0.49 to 0.31); .66; 0%; 0.0
Hematocrit change (%)	5.03 (4.5) vs 6.25 (2.5)	7.625±5.29 vs 9.73±9.41	NR	NR	−2.6±4.3 vs −2.5±5.1	NR	NR	NA	−0.29 (−2.15 to 1.58); .76; 34%; 0.9
Hemoglobin change (g/dL)	NR	NR	1.1±0.5 vs 1.14±0.5	NR	NR	NR	NR	NA	NC
Postinfusion plasma ionized calcium (mmol/L)	NR	1.39±0.0979 vs 1.17±0.04	1.1±0.17 vs 0.89±0.16	NR	1.30±0.05 vs 1.18±0.03	1.06±0.32 vs 1.04±0.20	NR	NA	**0.16 (0.08 to 0.23); <.001; 93%; 0.0**
Change in ionized calcium (mmol/L)	NR	0.20±0.20 vs −0.02±0.044	NR	NR	0.09±0.06 vs −0.02±0.04	NR	NR	NA	**0.16 (0.06 to 0.27); <.001; 92%; 0.01**
Cardiac arrhythmias	3/20 (15.0) vs 3/20 (15.0)	1/60 (1.7) vs 6/60 (10.0)	NR	NR	NR	NR	NR	4/80 vs 9/80	0.48 (0.08 to 2.69); .402; 47%; 0.775
Burning or discomfort at the intravenous site	1/20 (5.0) vs 0/20 (0.0)	1/60 (1.7) vs 3/60 (5)	0/183 (0.0) vs 0/184 (0.0)	NR	NR	NR	NR	2/263 vs 3/264	0.74 (0.14 to 3.88); .73; 0%; 0.00
5-min Apgar score	NR	NR	NR	NR	NR	8.90±0.54 vs 8.96±0.18	NR	NA	NC
Umbilical artery pH	NR	NR	NR	7.32±0.05 vs 7.32±0.02	NR	7.33±0.04 vs 7.31±0.05	NR	NA	0.008 (−0.01 to 0.02); .41; 51.3%; 0.00
NICU	NR	NR	NR	11/50 (22.0) vs 12/50 (24.0)	NR	NR	NR	11/50 vs 12/50	NC

RR, relative risk; MD, mean difference; RoM, ratio of means; NR, not reported by original study; NA, not available; NT, not transformable from median to mean; NC, not calculable; NICU, neonatal intensive care.

Data are mean±SD or n (%).

Bold indicates significance.

* Uterine tone measured by numerical score or binary outcome.

*Boers. Peri-delivery Calcium Administration. O&G Open 2026*.

The risk-of-bias assessment is presented in Appendix 3 (https://links.lww.com/AOG/E765). Of the seven included studies, five were judged to be at low risk of bias, one was judged as having some concerns, primarily because of the absence of a clinical trial registration, and one study by Yaqub et al^28^ was rated at high risk of bias, mainly because of insufficient reporting on outcome assessment and blinding procedures. Because fewer than 10 studies were included in this meta-analysis, assessment of publication bias was not feasible.

The GRADE analysis is presented in Appendix 4 (https://links.lww.com/AOG/E765). The certainty of evidence was high for postpartum blood loss. In contrast, the certainty of evidence for the need for additional uterotonics was rated moderate as a result of downgrading for inconsistency because there was moderate heterogeneity across studies (*I*^2^=46%).

## DISCUSSION

Postpartum hemorrhage is a major threat to maternal health worldwide. The increasing prevalence attributable to uterine atony underscores the need to explore additional uterotonic agents to minimize postpartum blood loss, especially given that current options have limitations. In this systematic review and meta-analysis of six RCTs and one quasi-randomized trial, we found that peri-delivery administration of calcium supplementation was associated with a reduction in postpartum blood loss. In addition, the pooled data suggest a lower need for additional uterotonics among women who received calcium supplementation.

To the best of our knowledge, this is the first systematic review and meta-analysis to synthesize evidence on peripartum calcium supplementation and postpartum bleeding outcomes. Therefore, our findings can be compared only with those of individual trials. Most of the included studies showed a reduction in postpartum blood loss after calcium supplementation. However, one trial conducted by Farber et al^26^ observed a nonsignificant increase in blood loss among participants receiving 400 mg calcium chloride compared with placebo (634±180 mL vs 588±161 mL). This finding may be explained by the relatively low dose of calcium chloride used, particularly compared with the 1-g doses administered in the studies by Ansari et al.^22,23^ In addition, this study enrolled healthy patients at low risk undergoing scheduled cesarean delivery. Because these participants were unlabored, the uterotonic response to oxytocin may have masked any additive effect of calcium chloride on uterine contractility.

Across the included studies, both the dose and formulation of calcium supplementation varied. It is important to note that the amount of ionized (free) calcium differs across calcium formulations: 1 g calcium chloride contains 272 mg elemental calcium, whereas 1 g calcium gluconate contains 92 mg elemental calcium.^31^ Both Abirami et al^27^ and Agarwal et al^27^ administered only 1 g intravenous calcium gluconate. Nonetheless, they observed a small but statistically significant reduction in postpartum blood loss. These effects may be attributed to low baseline ionized calcium levels in both studies. Although normal ionized calcium concentrations range from 1.16 to 1.31 mmol/L, baseline levels in all study groups were below this range.^20^ In addition, both studies were conducted in India, where hypocalcemia is more prevalent. In another Indian study, Yadav et al^25^ administered an even lower intrapartum dose of calcium supplementation of 500 mg calcium gluconate (equivalent to 46.5 mg elemental calcium) and did not measure ionized calcium levels. Instead, total serum calcium levels were measured, and baseline calcium levels in both study groups were below the normal range (8.50–10.50 mg/dL), suggesting that ionized calcium levels may also have been low.^32^ Moreover, many participants received magnesium supplementation, a calcium antagonist, and some received nifedipine, a calcium channel blocker, which likely further decreased intracellular calcium availability. Lower baseline calcium levels or simultaneous interventions affecting calcium physiology may explain why studies with lower calcium doses^24–27^ appeared to demonstrate an effect in these settings. Patients with lower calcium levels may even experience greater benefit from intrapartum calcium supplementation. Taken together, differences in calcium formulations, elemental dose, and baseline ionized calcium levels across studies may have contributed to the observed variation in postpartum blood loss outcomes.

Calcium supplementation is hypothesized to improve uterine contractility and may therefore reduce postpartum blood loss attributable to uterine atony rather than bleeding attributed to other causes. Only one study, conducted by Ansari et al,^23^ evaluated the effect of calcium supplementation in patients with uterine atony. Among 120 laboring patients, the median blood loss was not statistically different between the intervention and control arms (840 mL vs 1,051 mL, mean reduction 211 mL, 95% CI, −33 to 410). However, in the planned subgroup analysis (n=79) excluding patients with nonatonic bleeding, calcium reduced blood loss by 350 mL (95% CI, 159–515). Although the other studies included in this review did not specifically assess calcium supplementation in patients with uterine atony, most attempted to enroll patients at increased risk of atony such as women undergoing intrapartum cesarean delivery or those exposed to oxytocin. In contrast, the studies by Farber et al^26^ and Agarwal et al^27^ were the only ones to enroll patients at low risk undergoing unlabored scheduled cesarean deliveries, a population known to be at low risk of uterine atony. This low baseline risk may further explain the limited effect on postpartum blood loss observed in the trial by Farber et al.

Beyond clinical trials, translational research supports the biologic rationale that altered calcium homeostasis in uterine smooth muscle cells underlies uterine atony. Uterine atony is challenging to study because there are no in vivo or in vitro models.^33^ However, Ansari et al^33^ conducted a prospective translational study in which they demonstrated an association between the clinical diagnosis of uterine atony and impaired oxytocin-induced calcium signaling in uterine smooth muscle cells. They showed that patients with uterine atony have a decreased oxytocin-induced rise in intracellular calcium concentration compared with women with normal tone (22% vs 72%). This suggests an impaired calcium–contractility pathway. Whether supplementing exogenous calcium can (partially) overcome this impaired intracellular calcium signaling remains unknown. Our findings also align with the clinical perspectives summarized in the editorial by Ansari and Smiley,^34^ which highlighted the biologic plausibility of calcium as a uterotonic and compared the outcomes reported by Ansari et al^22,23^ and Abirami et al.^24^

This study has several strengths, including a preregistered and published PROSPERO protocol and a comprehensive systematic search with clear inclusion and exclusion criteria without language, geographic, or date restrictions. Therefore, we included patients across diverse countries, including the United States, India, and Pakistan. We included only RCTs to minimize confounding. In addition, we attempted to account for between-study differences by calculating the heterogeneity using *I*^2^ and τ^2^. Finally, dual study screening and data extraction minimized the risk of errors. In addition to assessing the risk of bias, we graded the certainty of the evidence using the GRADE approach.

However, the limitations of this study should be acknowledged. First, the studies included in this meta-analysis were few and generally had small sample sizes. In addition, many studies did not report the same outcomes, which limited our ability to draw conclusions across all variables. Consequently, sensitivity and subgroup analyses could not be performed. Only one study^23^ was powered to detect differences in blood loss because it was the primary outcome. In addition, postpartum blood loss is typically skewed, making means less appropriate for comparison than medians. Moreover, estimated blood loss is prone to visual estimation error.^35^ Quantitative blood loss measurement may improve accuracy and is recommended over visual estimation. However, it also has limitations, including inaccurate results attributable to technical problems and contamination with amniotic or irrigation fluids, and has not been shown to improve clinical outcomes.^35,36^

Furthermore, several outcomes such as the use of second-line uterotonics or the clinical diagnosis of uterine atony are inherently subjective. However, the double-blinded randomized design of the included studies likely minimized bias arising from subjective outcome assessment. Nevertheless, the reduced need for additional uterotonics remains a clinically relevant exploratory outcome that may suggest a uterotonic effect of calcium. Moreover, there was considerable heterogeneity across studies in terms of patient population, calcium formulations, and calcium doses. Most studies used calcium preparations with relatively low elemental calcium, and not all patients were at risk for uterine atony. These factors may have resulted in a modest effect size. Although the observed reduction in postpartum blood loss may not be clinically significant, it suggests that calcium has uterotonic properties. Indeed, the true effect may be larger in those who are at higher risk for uterine atony or when higher doses are used. However, given the limitations of this meta-analysis, the findings should be interpreted with caution, and future research should specifically evaluate populations at higher risk and dosing strategies.

Potential harms of calcium supplementation should be also considered. Intravenous calcium administration may cause adverse effects, including extravasation leading to local tissue necrosis, hemodynamic changes, and cardiac conduction abnormalities.^37,38^ Although calcium supplementation was well tolerated across the included studies, these studies were not powered to detect adverse events, and adverse event reporting was inconsistent across studies. Therefore, the safety of peri-delivery calcium administration requires further investigation in larger RCTs. Administration through slow, controlled infusion and patient monitoring remain important.

The strength of a systematic review depends on the quality of the included studies. In this meta-analysis, most of the studies were rated as being at low risk of bias, with one study rated as having some concerns and one study judged to be at high risk of bias.^28^ It is important to note that this study did not contribute to the analyses of postpartum blood loss or the need for additional uterotonics. Consistent with the GRADE assessment, the certainty of the evidence was high for postpartum blood loss but moderate for the need for additional uterotonics.

Taken together, the results of this meta-analysis suggest that peripartum exogenous calcium administration may be a promising adjunctive strategy to reduce postpartum blood loss and lower the need for additional uterotonics. However, the observed effect size was modest, and the evidence was limited by the small number of available studies and heterogeneity in calcium formulations and dosing. Therefore, the outcomes of this review should be interpreted with appropriate caution. Given the favorable characteristics of calcium and these encouraging preliminary findings, further investigation is warranted. Larger, adequately powered RCTs are needed to assess the effect of intrapartum calcium supplementation on postpartum bleeding outcomes and to evaluate higher calcium doses to confirm efficacy and determine optimal dosing for patients at increased risk for uterine atony.
